# Expanding the CRISPR/Cas9 toolkit for *Pichia pastoris* with efficient donor integration and alternative resistance markers

**DOI:** 10.1002/jcb.26474

**Published:** 2017-12-26

**Authors:** Astrid Weninger, Jasmin E. Fischer, Hana Raschmanová, Claudia Kniely, Thomas Vogl, Anton Glieder

**Affiliations:** ^1^ Institute of Molecular Biotechnology Graz University of Technology Graz Austria; ^2^ Bisy e.U. Wetzawinkel, Hofstätten/Raab Austria; ^3^ Department of Biotechnology University of Chemistry and Technology Prague Prague Czech Republic

**Keywords:** CRISPR/Cas9, homologous recombination, *KU70*, marker recycling, *Pichia pastoris*, resistance markers

## Abstract

*Komagataella phaffii* (syn. *Pichia pastoris*) is one of the most commonly used host systems for recombinant protein expression. Achieving targeted genetic modifications had been hindered by low frequencies of homologous recombination (HR). Recently, a CRISPR/Cas9 genome editing system has been implemented for *P. pastoris* enabling gene knockouts based on indels (insertion, deletions) via non‐homologous end joining (NHEJ) at near 100% efficiency. However, specifically integrating homologous donor cassettes via HR for replacement studies had proven difficult resulting at most in ∼20% correct integration using CRISPR/Cas9. Here, we demonstrate the CRISPR/Cas9 mediated integration of markerless donor cassettes at efficiencies approaching 100% using a *ku70* deletion strain. The Ku70p is involved in NHEJ repair and lack of the protein appears to favor repair via HR near exclusively. While the absolute number of transformants in the Δ*ku70* strain is reduced, virtually all surviving transformants showed correct integration. In the wildtype strain, markerless donor cassette integration was also improved up to 25‐fold by placing an autonomously replicating sequence (ARS) on the donor cassette. Alternative strategies for improving donor cassette integration using a Cas9 nickase variant or reducing off targeting associated toxicity using a high fidelity Cas9 variant were so far not successful in our hands in *P. pastoris*. Furthermore we provide Cas9/gRNA expression plasmids with a Geneticin resistance marker which proved to be versatile tools for marker recycling. The reported CRSIPR‐Cas9 tools can be applied for modifying existing production strains and also pave the way for markerless whole genome modification studies in *P. pastoris*.

## 1 INTRODUCTION

Within a few years, genome engineering by CRISPR/Cas9 (clustered regularly interspaced short palindromic repeats/CRISPR associated protein 9) became one of the most valuable tools in biosciences.^1^, ^2^, ^3^ This method facilitates targeted, programmable genome modifications and provides major advantages over classical knock‐out‐, knock‐in‐approaches as well as alternative genome engineering strategies. Besides its potency in basic research for studying disease conditions,^4^, ^5^, ^6^, ^7^ CRISPR/Cas9 was also used for host (strain) engineering for biotechnological production processes.^8^, ^9^, ^10^ Prior to CRISPR/Cas9, highly efficient genome manipulations were difficult to achieve in many organisms. Gene targeting with conventional knock‐out/knock‐in cassettes containing homologous sequences depended on the organisms amenability for homologous recombination and homology cassettes were predominantly integrated ectopically.^11^, ^12^ In addition, the replacement of DNA regions or the insertion of markers (or other disruption cassettes) changes the context of the genome sequence and can influence neighboring genes and their expression. This is especially relevant for maintaining the functional fidelity of tightly packed microbial genomes, where adjacent open reading frames are sometimes only separated by very short DNA stretches. Genome engineering methods available prior to the advent of CRISPR/Cas9 such as Zinc‐finger nucleases or TALENs^12^, ^13^, ^14^ required laborious protein engineering to reprogram the targeting locus. CRISPR/Cas9 allows to easily introduce targeted strand breaks in almost any desired genomic locus and can easily be reprogrammed. The DNA endonuclease Cas9 is guided by a short guide RNA (gRNA, or single guide RNA, sgRNA) to introduce a double strand break (DSB) at genomic regions complementary to the gRNA sequence. The strand breaks are sealed by the cell's endogenous repair machinery, allowing the introduction of various genomic modifications.^15^ Reprogramming CRISPR/Cas9 and thereby targeting different loci is performed by changing a 20 bp sequence of the gRNA. Besides a growing list of organisms, in which CRISPR/Cas9 systems were successfully applied for genome engineering,^1^, ^9^, ^16^, ^17^ also continuous improvements of the method itself have been reported. Particularly remarkable are high‐fidelity Cas9 enzymes (eSpCas9, SpCas9‐HF1), which operate almost exclusively at on‐target sites.^18^, ^19^ Thereby unwanted off‐targeting, one of the major drawbacks of CRISPR/Cas9, could be reduced.

Due to its potent secretion of pure protein, less extensive glycosylation and high cell density growth *P. pastoris* (*syn. Komagataella phaffii*) is one of the most commonly used eukaryotic expression hosts.^20^, ^21^ While molecular mechanisms in the “classic” yeast *S. cerevisiae* are well‐studied, similar information is widely lacking in *P. pastoris*.^22^ A key advantage of *S. cerevisiae* has been the ease to perform genome modifications due to its remarkably efficient homologous recombination machinery. For *P. pastoris*, and also other non‐conventional yeasts, the deletion of genes and the resulting studies of gene functions are more challenging. Deletion cassettes for targeted gene disruption are often randomly inserted in the genome by ectopic integration (with correct targeting rates of <1‐30%^23^), requiring laborious screening procedures to identify correct knockout strains. In order to increase the homologous recombination (HR) frequencies in *P. pastoris* for classical HR experiments, a Δ*ku70* was generated previously, which lacks the gene coding for the Ku70 protein, a key player in the NHEJ mechanism.^23^ Using this strain, targeting efficiencies over 90% with knockout cassettes containing only 250 bp flanking homology arms had been obtained under selective conditions.^23^

The transformation of selection marker‐free fragments has yet not even been performed with the *P. pastoris* Δ*ku70* strain, because it would require intensive screening (since lack of selection after a transformation would result in outgrowth of unmodified cells that did not take up exogenous DNA). However, double HR of a (marker‐free) donor DNA cassette is required to completely delete a CDS or to replace it with a different sequence at a genomic locus. Targeted single and double strand break induced DNA repair can be used to increase the HR frequency by several orders of magnitude.^24^, ^25^, ^26^ When co‐transforming homology donor cassettes and CRISPR/Cas9 expression vectors an increase in the HR rate was observed for most yeast species, for which a CRISPR/Cas9 system has been developed.^27^, ^28^, ^29^, ^30^, ^31^ The increase in HR frequency due to CRISPR/Cas9 targeted cleavage also allowed the integration of marker‐less donor fragments.

**Figure 1:**
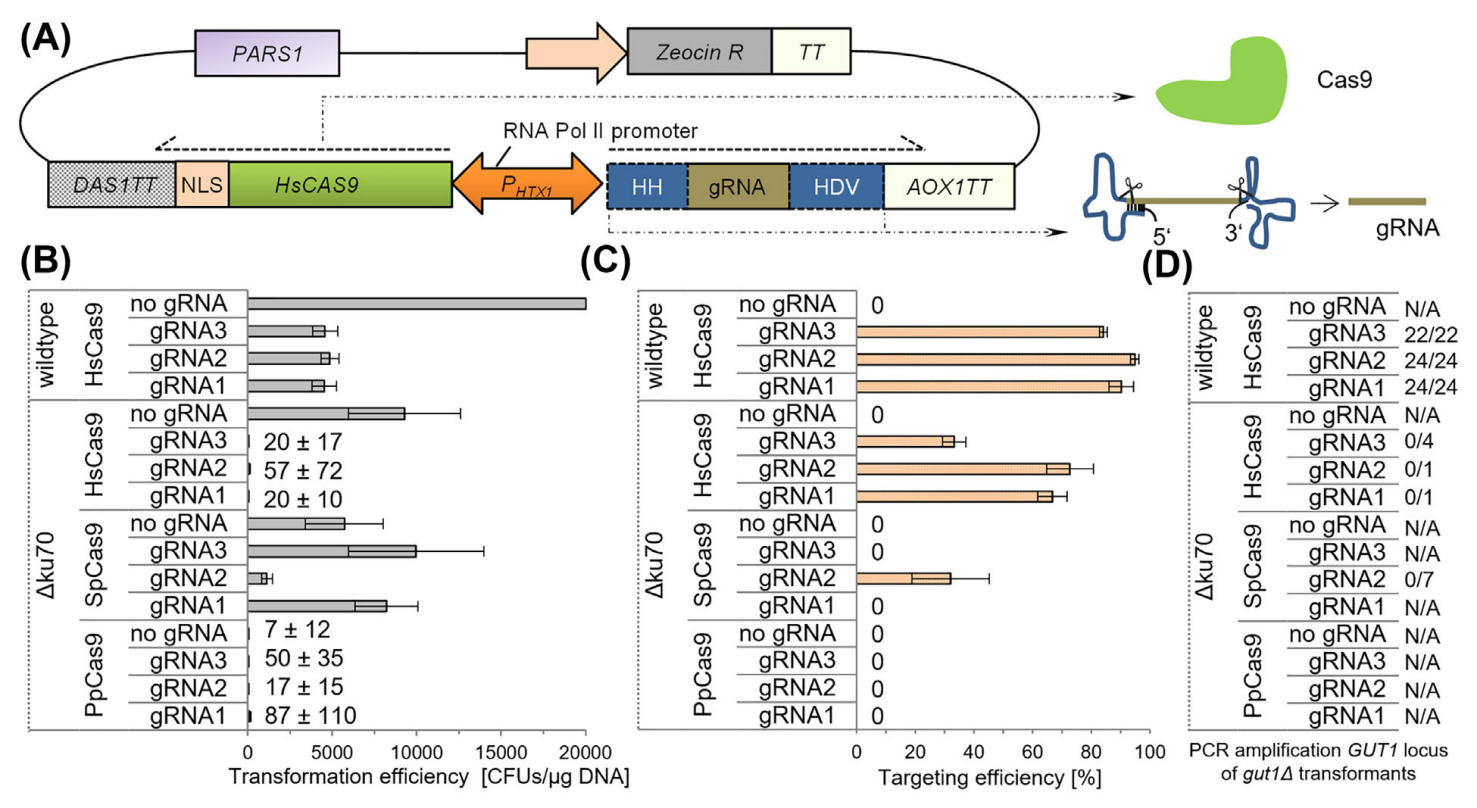
Functional Cas9/gRNA expression drastically decreases transformation efficiencies in *P. pastoris* Δ*ku70* compared to the wildtype strain. A, Schematic illustration of the Cas9 and gRNA expression strategy successfully calibrated previously^32^ and used as the basis for this study. A human codon optimized *CAS9* gene fused to a C‐terminal SV40 nuclear localization sequence and the gRNA flanked by hammerhead (HH) and hepatitis delta virus (HDV) ribozymes are expressed from an episomal plasmid (carrying the PARS1 autonomously replicating sequence and a Zeocin resistance marker). The ribozymes are transcribed and auto‐catalytically cleave themselves resulting in a gRNA without additional sequences from the 5′ or 3′ UTR of the promoter/terminator used. B‐D, *P. pastoris* CBS 7435 Δ*ku70* and wildtype^32^ strains were transformed with CRISPR/Cas9 plasmids containing either *PpCAS9*, *HsCAS9* or *SpCAS9* codon optimized sequences and gRNAs to target *GUT1*. Mean values and standard deviations of biological triplicates are shown. B,Transformation efficiencies of *P. pastoris* CBS 7435 Δ*ku70* and wildtype with CRISPR/Cas9 plasmids. *P. pastoris* CBS 7435 wildtype results have been reported previously.^32^ HsCas9 (w/o gRNA) yielded >20 000 *P. pastoris* wildtype transformants and partly a cell lawn, suggesting that the nuclease is barley active having no detrimental effects on cell growth. The functional expression of HsCas9 and different gRNA yielded a reduction of the transformation efficiencies in *P. pastoris* wildtype. The CRISPR/Cas9 systems were even more lethal for the *P. pastoris* Δ*ku70* strain, where the transformation efficiencies decreased drastically. C, *GUT1* targeting efficiencies of *P. pastoris* CBS 7435 *Δku70* and wildtype with CRISPR/Cas9 plasmids. The targeting efficiencies were reduced compared to the *P. pastoris* wildtype strain. Among the different Cas9 different codon optimized sequences, HsCas9 was most effective in *P. pastoris Δku70*. D, Amplification of the *GUT1* locus upon CRISRP/Cas9 cleavage. NHEJ‐mediated indel mutations were obtained in the *P. pastoris* wildtype strain. The indel mutations were verified by sequencing.^32^ The amplification of the *GUT1* in the *P. pastoris* Δ*ku70* strain was not possible with the same primers used for the *P. pastoris* wildtype strain (see S3)

A highly efficient CRISPR/Cas9 system was also implemented for the methylotrophic yeast *P. pastoris*.^32^ A human codon optimized *CAS9* nuclease and ribozyme‐flanked gRNAs were expressed under the control of a bidirectional, constitutive RNA polymerase II promoter (Figure 1A). The ribozymes were required for the processing and functional production of gRNAs without additional 5′/3′ sequences (stemming from the 5′/3′ UTRs of the promoter/terminators used). The CAS9/gRNA expression cassettes were provided on an episomal plasmid and were cured by growth on non‐selective media. In *P. pastoris* the CRISPR/Cas9 induced double strand breaks are repaired by the cellular NHEJ machinery, resulting in the formation of short insertions and deletions (indels) in the coding sequence (CDS), and frameshift mutations. The *P. pastoris* CRISPR/Cas9 system enables gene deletions at targeting rates approaching 100% and also the simultaneous deletion of different genes by using multiple gRNAs.^32^ However, specifically integrating donor cassettes via homologous recombination (HR) for replacement studies had proven difficult resulting in low efficiencies in correct integration using CRISPR/Cas9 in *P. pastoris*.

Here, we expanded the existing CRISPR/Cas9 genome engineering system for *P. pastoris* by overcoming these previous limitations. We demonstrate the integration of marker‐free donor cassettes at efficiencies approaching 100% using a *ku70* deletion strain previously reported.^23^ Alternatively, in *P. pastoris* wildtype strains integration rates for marker‐free donor cassettes of 50% were obtained by using stability donor cassettes with autonomously replicating sequences (ARSs). ARSs are typically placed on plasmids to allow episomal expression, which is beneficial for the production of selected proteins.^32^ Depending on the ARS multiple copies of the plasmids are present in the cell (PARS1, 10 copies) and the plasmids are maintained in the presence of selective pressure.^33^ Another previously reported strategy to increase the HR frequencies is the expression of the Cas9 nickase, which was also evaluated in this study. This Cas9 variant creates single strand breaks due to the inactivation of one of its catalytic domains via the introduction of a point mutation.^34^, ^35^ By introducing single strand breaks an increase in donor recombination was demonstrated by^36^, ^37^ in mammalian cells. However, the use of the Cas9 nickase did so far not show clear advantages in our hands in *P. pastoris*.

## 2 MATERIALS AND METHODS

### 2.1 Chemicals

Enzymes were obtained from Thermo Fisher Scientific, Vienna, Austria. D(+)‐biotin was obtained from Sigma‐Aldrich, Vienna, Austria. Difco yeast nitrogen base w/o amino acids, Bacto tryptone and Bacto yeast extract were obtained from Becton Dickinson, Schwechat, Austria. Zeocin was obtained from Thermo Fisher Scientific. Other chemicals were purchased from Carl Roth, Karlsruhe, Germany. Oligonucleotides were ordered from Integrated DNA Technologies, Leuven Belgium, see supplementary Table S1 for the sequences.

### 2.2 Strains and constructs

Yeast strains in this study were based on the *P. pastoris* wildtype strain CBS 7435 (identical to NRRL Y‐11430 and ATCC 76273). For some experiments a closely related *P. pastoris* BSYBG11 mutS strains (NRRL Y‐11430 Δ*aox1* derivatives lacking episomal killer plasmids,^38^ obtained from bisy e.U., Austria) were used. The plasmids used for the expression of the CRISPR/Cas9 constructs are based on the *E.coli/P. pastoris* shuttle vector pPpT4_GAP (Genbank accession number: JQ519692^23^). The generation of several CRISPR/Cas9 constructs had been described in the paper of Weninger et al.^32^ Here we used only the most efficient designs (number 81‐83^32^). All plasmids were sequenced by Sanger sequencing. Maps of key plasmids generated in this study are provided in supplementary File S2. Transformations and screenings were performed as described by Weninger et al.^32^

#### 2.2.1 Co‐transformation of CRISPR/Cas9 plasmids and donor cassettes in *P. pastoris* CBS7435 Δku70

The *P. pastoris* CBS7435 *Δku70* strain reported previously was used.^23^ In short, a knock‐in and partial deletion into the *KU70* gene had been performed and in addition the start codon had been also modified to cause a frameshift. The correctness of the modification had been verified and the strain comprehensibly characterized.^23^, ^39^ The generation of the donor cassettes had been described previously.^32^ The donor cassette bearing PARS1 was generated as follows: pJET1.2 blunt‐GUT1 (CloneJET PCR Cloning Kit, Thermo Fisher Scientific), consisting of the pJET1.2 backbone and the 5′ upstream fused to the 3′ downstream regions of the *GUT1* locus, was linearized using *Pst*I. PARS1 was amplified from the plasmid pPpT4_HTX1‐PARS1‐HsCas9^32^ using the primer pair Pjet‐ARS‐fw/Pjet‐ARS‐rv. The PARS1 PCR fragment was cloned into the vector backbone by assembly cloning.^40^ One hundred nanograms of the purified circular donor cassettes and 1 μg of the circular or linearized, purified donor cassettes (*Hin*dIII linearized pJET1.2 blunt‐GUT1‐PARS1) were used for the *P. pastoris* transformation. The primers GUTout3prR1 and 5prGUT1_fwd were used for the amplification of the *GUT1* locus and the primer PGUTseq2 was used for sequencing. In case of *P. pastoris Δku70* transformants, which displayed a growth defect on glycerol upon the transformation with circular CRISPR/Cas9 plasmids, the primers GUT1‐3‐fw and GUTout3prR1 were used for the amplification of the *GUT1* locus and the primers GUT1‐438‐seq‐fw and GUT1‐525‐seq‐rv were used for sequencing.

#### 2.2.2 Constructs for DNA deletion at the DAS1/DAS2 locus

The plasmid pPpT4_HTX1‐PARS1‐HsCas9^32^ was linearized with *Not*I. The gRNAs we ordered on synthetic double stranded DNA fragments (gBlocks, Integrated DNA technologies, RZ‐DAS1/2‐gRNA1‐RZ and RZ‐DAS1/2‐gRNA2‐RZ) and cloned into the linearized plasmid by assembly cloning. One hundred nanograms plasmid DNA were used for the *P. pastoris* transformations. Randomly picked transformants were cultivated in 96‐deep well plates (96‐DWPs, 250 μL YPD‐Zeocin [50 μg/mL]) and stamped on agar plates with either glucose (BMD1) or methanol (BMM1) as carbon source. The primers Das2_rev and Das1_fw were used for the amplification and for sequencing of the *DAS1/DAS2* locus, the primers Das2_rev and Das2_fw were used for the amplification of the *DAS2* locus, and the primers Das1_rev and Das1_fw were used for the amplification of the *DAS1* locus.

#### 2.2.3 CRISPR/Cas9 nickase constructs

For the construction of vectors bearing *Hs*Cas9 nuclease, the plasmid pPpT4_HTX1‐PARS1‐HsCas9^32^ was linearized with *Not*I. The gRNAs we ordered on synthetic double stranded DNA fragments (gBlocks, Integrated DNA technologies, RZ‐GUT1‐gRNA0‐RZ, and RZ‐GUT1‐gRNA4‐RZ) and cloned into the linearized plasmid by assembly cloning. For the construction of vectors bearing *Hs*Cas9 nickase, the plasmid pPpT4_HTX1‐PARS1^32^ was linearized with *Eco*RI. *Hs*Cas9 was amplified from the plasmid pPpT4_HTX1‐PARS1‐HsCas9 using the primers DAS1TT‐hsCas9‐rv and HsCas9‐D10A‐fw, containing the point mutation 10D → A (codon GAT to GCT). The PCR amplicon and the vector backbone were assembled to generate pPpT4_HTX1‐PARS1‐HsCas9‐nickase. pPpT4_HTX1‐PARS1‐HsCas9‐nickase was linearized with *Not*I and the gRNAs GUT1‐gRNA0 and GUT1‐gRNA4 were cloned into the backbone.

#### 2.2.4 Construction of CRISPR/Cas9 plasmid bearing high fidelity HsCas9

The vector pPpT4_GAP1‐PARS1‐Zeo^32^ was cleaved with *Pci*I and *Not*I to remove the *GAP* promoter (*P_GAP_*). A fragment containing the *DAS1TT* terminator, the SV40 nuclear localization signal and a part of *Hs*Cas9 coding sequence (bp 2780‐4104) was PCR amplified from plasmid pPpT4_pHTX1‐HsCas9‐RZ‐GUT1‐gRNA1^32^ using primers P_AOX1_syn_pUCORI_Gibs_fv and HsCas9_rev. A fragment containing a part of *Hs*Cas9 coding sequence (bp 1‐2255), the *P_HTX1_* bidirectional promoter, the hammerhead ribozyme (HH), GUT1‐gRNA1, and the hepatitis delta virus ribozyme (HDV) was also PCR amplified from the plasmid pPpT4_pHTX1‐HsCas9‐RZ‐GUT1‐gRNA1 using primers HsCas9_fv and HDV_AOX1TT_Gibs_rev. The part of *Hs*Cas9 coding sequence, in which four amino acids should be exchanged according to Kleinstiver et al^18^ in order to prepare the off‐target free version of *Hs*Cas9 (497N → A, 661R → A, 695Q → A, 926Q → A), was ordered as synthetic double stranded DNA (gBlock_hf‐HsCas9). GCT was used as codon for alanine at all four positions. The two PCR fragments and the synthetic double stranded DNA fragment were cloned in the vector backbone by assembly cloning to form the plasmid pPpT4_pHTX1‐hf‐HsCas9‐GUT1‐gRNA1 (supplementary File S2). For the genotypic characterization the *GUT1* locus was amplified from colonies applying the Phire Plant Direct PCR Master Mix (Thermo Fisher Scientific, Vienna, Austria) with primers 3UTRGUTR and seq‐pGUT1‐332..308‐fwd. Primer seq‐pGUT1‐332..308‐fwd was used for sequencing.

#### 2.2.5 CRISPR/Cas9 plasmids bearing a geneticin resistance cassette

The plasmid pPpT4_HTX1‐PARS1‐HsCas9^32^ was cut with the restriction enzymes *Xba*I and *Smi*I to remove the Zeocin resistance marker cassette. Accordingly, the plasmid pPpT4_S_Kan (Genbank accession number: JQ19694^23^) was cut with the restriction enzymes *Xba*I and *Smi*I to obtain a fragment containing the Geneticin resistance marker cassette. The backbone and the insert were assembled by ligation with the T4 DNA ligase to create pPpT4_HTX1‐PARS1‐HsCas9‐Gen, supplementary File S2. pPpT4_HTX1‐PARS1‐HsCas9‐Gen was linearized with *Not*I and the gRNAs RZ‐GUT1‐gRNA1‐RZ, RZ‐GUT1‐gRNA2‐RZ, RZ‐GUT1‐gRNA3‐RZ to target *GUT1* as well as the gRNAs ZEO‐gRNA1, ZEO‐gRNA2, ZEO‐gRNA3 to target the Zeocin resistance gene, ordered on synthetic double stranded DNA fragments, were cloned into the plasmid by assembly cloning. The *P. pastoris* CBS7435 wildtype strain expressing enhanced green fluorescent protein (e*GFP*,^41^ Institute of Molecular Biotechnology (IMBT, Graz University of Technology, Austria) strain collection number: 8008) and the *P. pastoris* BSYBG11 mutS strains expressing glucose oxidase (*GOX*, accession number: AID16306.1, synthetic gene, codon optimized for *P. pastoris*, IMBT strain collection number: 8009), and *Candida antarctica* lipase B (*CALB*,^42^ synthetic gene, codon optimized for *P. pastoris*, IMBT strain collection number: 8010) from ectopically integrated expression cassettes in their genomes were transformed with 100 ng circular CRISPR‐Cas9 plasmid DNA. Random transformants were cultivated in 96‐DWPs containing 250 μL YPD‐Geneticin media (300 μg/mL Geneticin) to support maintenance of ARS plasmids. After 60 h the transformants were stamped on YPD, YPD‐Zeocin (50 μg/mL) and YPD‐Geneticin (300 μg/mL) agar plates to determine the targeting efficiency by phenotypic characterization.

## 3 RESULTS AND DISCUSSION

### 3.1 Increasing the efficiency of homology‐directed repair (HDR) in *P. pastoris*

#### 3.1.1 CRISPR‐Cas in ***P. pastoris Δku70*** allows integration of donor cassettes at near 100% efficiency

##### NHEJ‐repair appears strongly impaired using CRISPR/Cas9 in ***P. pastoris Δku70*** strain

As the CRISPR/Cas9 mediated integration of donor cassettes in *P. pastoris* wildtype strains was so far inefficient and in part even unsuccessful,^32^ we evaluated to combine the potency of the *P. pastoris Δku70* strain and CRISPR/Cas9 to increase the HR rates and to specifically enable the integration of marker‐less fragments. Prior to integration studies using homology donor cassettes, we characterized CRISPR/Cas9 mediated gene deletion by the double strand cleavage in *P. pastoris Δku70*. Due to the impaired NHEJ machinery of *P. pastoris Δku70* CRISPR/Cas9 expression might be toxic and/or lethal, even if homology donor fragments are co‐transformed. The *P. pastoris* wildtype and *Δku70* strains were transformed with CRISPR/Cas9 plasmids (Cas9 + gRNA) without homology donor cassettes (Figure 1B‐D). The CRISPR/Cas9 vectors bore ribozyme flanked gRNAs to target *GUT1* and *CAS9* sequences with different codon optimization (*P. pastoris* codon optimized *CAS9* [*PpCAS9*], *Streptococcus pyogenes CAS9* [*SpCAS9*], and a human codon optimized *CAS9* [*HsCAS9*]) under the control of a bidirectional promoter.^32^ In our previous study in the wildtype strain,^32^ it appeared that *Sp*Cas9 is poorly expressed, *Pp*Cas9 is expressed at too high levels and only *Hs*Cas9 is giving optimal expression permitting high genome editing efficiencies. Hence, we tested here again all three available Cas9 variants, to evaluate if for the *P. pastoris Δku70* strain a different expression level would be needed (for the wildtype strain only the established *Hs*Cas9 was included).

The simple *GUT1* locus was targeted as *GUT1* defective strains display a reduced growth phenotype on glycerol as carbon source compared to the wild type strain.^23^ Hence mutations in the *GUT1* coding sequence (CDS) could be easily detected by replica‐stamping the cells on glycerol plates. The transformation rates of the *Δku70* strain with the CRISPR/Cas9 plasmids were lower compared to the wildtype strain (*P. pastoris* wildtype: ∼5000 colony forming units [CFUs]/μg DNA, depending on the gRNA used^32^). Less than 60 *P. pastoris* Δ*ku70* CFUs/μg plasmid DNA were obtained with the constructs bearing either *Hs*Cas9 or *Pp*Cas9 and one of the three gRNAs (Figure 1B) yielding an almost 100‐fold reduction compared to the transformation of wildtype strain (expressing *HsCAS9*). None of the *Pp*Cas9 transformants had growth deficiencies on glycerol plates indicating that no double strand break followed by error prone NHEJ‐repair took place at the target locus (Figure 1C). 33‐73% of the clones bearing *Hs*Cas9 displayed a reduced growth phenotype on glycerol. The targeting efficiency of *Hs*Cas9 depended on the gRNA used and was reduced compared to the *P. pastoris* wildtype strain (where 87‐94% of transformants could not grow on glycerol), confirming the expectation that the Ku70 protein is involved in the error prone repair of double strand breaks caused by the targeted Cas9 mediated double strand break. The number of clones obtained after the transformation with *Sp*Cas9 differed depending on the gRNA sequence. Transformations with *Sp*Cas9 and gRNA1 or gRNA3 resulted in more than 10 000 CFUs/μg DNA. None of the transformants showed reduced growth on glycerol. Approximately 1500 CFUs/μg DNA were obtained, when a plasmid bearing *Sp*Cas9 and gRNA2 had been transformed. Thereof 32% of the transformants had a reduced growth phenotype on glycerol. This result suggests that, in combination with the *SpCAS9* sequence, gRNA1 and gRNA3 are not functional or at least less functional than gRNA2. At the same time, functional gRNA expression appears linked to reduced transformation rates, possibly due to double strand breaks that could not be repaired efficiently in the NHEJ deficient *Δku70* strain. This notion is supported by the results of *HsCAS9* in the *Δku70* strain were even higher targeting efficiencies correlated with a higher reduction in transformation rates. These observations were not surprising considering the fact that translocations are common, when proteins involved in NHEJ are missing.^43^ Also in the absence of gRNAs a reduction in the targeting efficiency was observed for the *P. pastoris* wildtype^32^ and *Δku70* strain, when expressing *Hs*Cas9 and *Pp*Cas9, which indicates a toxic effect due to the abundance of high levels of the Cas9 protein localized in the nucleus (which may interfere with cellular processes such as DNA replication, transcription and transcription factor binding). The *Δku70* strain appears to be even more susceptible to this effect (than the wildtype strain) possibly owing to its reduced stress resistance.^23^

Genomic DNA of growth deficient and normally growing transformants of all constructs was isolated. Interesting, we were not able to amplify the *GUT1* locus of in *Δgut1* mutants with primers, which had been used for the amplification of genomic DNA isolated from *P. pastoris* wildtype CRISPR/Cas9 transformants (Figure 1D).

These results strongly suggest that NHEJ is practically completed abolished in the *Δku70* strain: First, the transformation rates were drastically reduced compared to the wildtype strain (∼100‐fold) when coexpressing gRNAs, indicating that DSBs introduced by Cas9 cannot be repaired and are lethal. Expression of Cas9 without gRNAs in the Δ*ku70* strain shows only a moderate decrease in the transformation rate to about half of the wildtype strain. This effect may be attributable to different amenabilities of the Δ*ku70* strain to transformations or different tolerance to stress caused from expression of substantial amounts of the heterologous Cas9 protein. Second, none of the *P. pastoris* Δ*ku70* Δ*gut1* strains obtained tested positive for NHEJ mutations (Figure 1D). While we could PCR amplify the targeted *GUT1* locus in all 70 transformants for the wildtype strain, we could not get PCR products for any of the 11 clones Δ*ku70* transformants with the primers used for the amplification of the *GUT1* locus of wildtype transformants. However, when using a forward primer, which bound further upstream of the Cas9 cleavage site, the amplification of the *GUT1* locus was possible (supplementary Figure S3). Sanger sequencing revealed that DNA fragments of approximately 600 bp upstream of the Cas9 cleavage position were deleted in the *P. pastoris* Δ*ku70* strain. This correlates to findings in various eukaryotic hosts, where the deletion of *KU* genes resulted in extended DNA degradation and increased deletion sizes upon double strand cleavage.^44^ These results suggest, that most probably the DSB in the Δ*ku70* was repaired by other means than NHEJ, for example translocation events taking place, which caused the rearrangement with non‐homologous parts of the chromosomes, or by microhomology‐mediated end joining (MMEJ).^45^

*P. pastoris* Δ*ku70* transformants with wildtype like growth did not contain a mutation in the target sequence. CRISPR/Cas9 expression has already been described in various NHEJ‐deficient organisms, mainly in combination with homology donor fragments.^28^, ^46^, ^47^ Similar as observed for *P. pastoris* Δ*ku70*, CRISPR/Cas9 expression (*CAS9* + gRNA) in the oleaginous yeast *Yarrowia lipolytica* Δ*ku70* and Δ*ku70*/Δ*ku80* strains yielded reduced targeting and transformation efficiencies.^46^ Based on these results, CRISPR/Cas9 vectors bearing ribozyme flanked gRNAs to target *GUT1* and *HsCAS9* were selected for the co‐transformation experiments with homology donor cassettes, because the highest targeting efficiencies/functionalities were obtained with these constructs in the Δ*ku70* strain.

##### CRISPR/Cas9 + donor DNA co‐transformation in *P. pastoris* Δ*ku70* increases HDR

**Figure 2:**
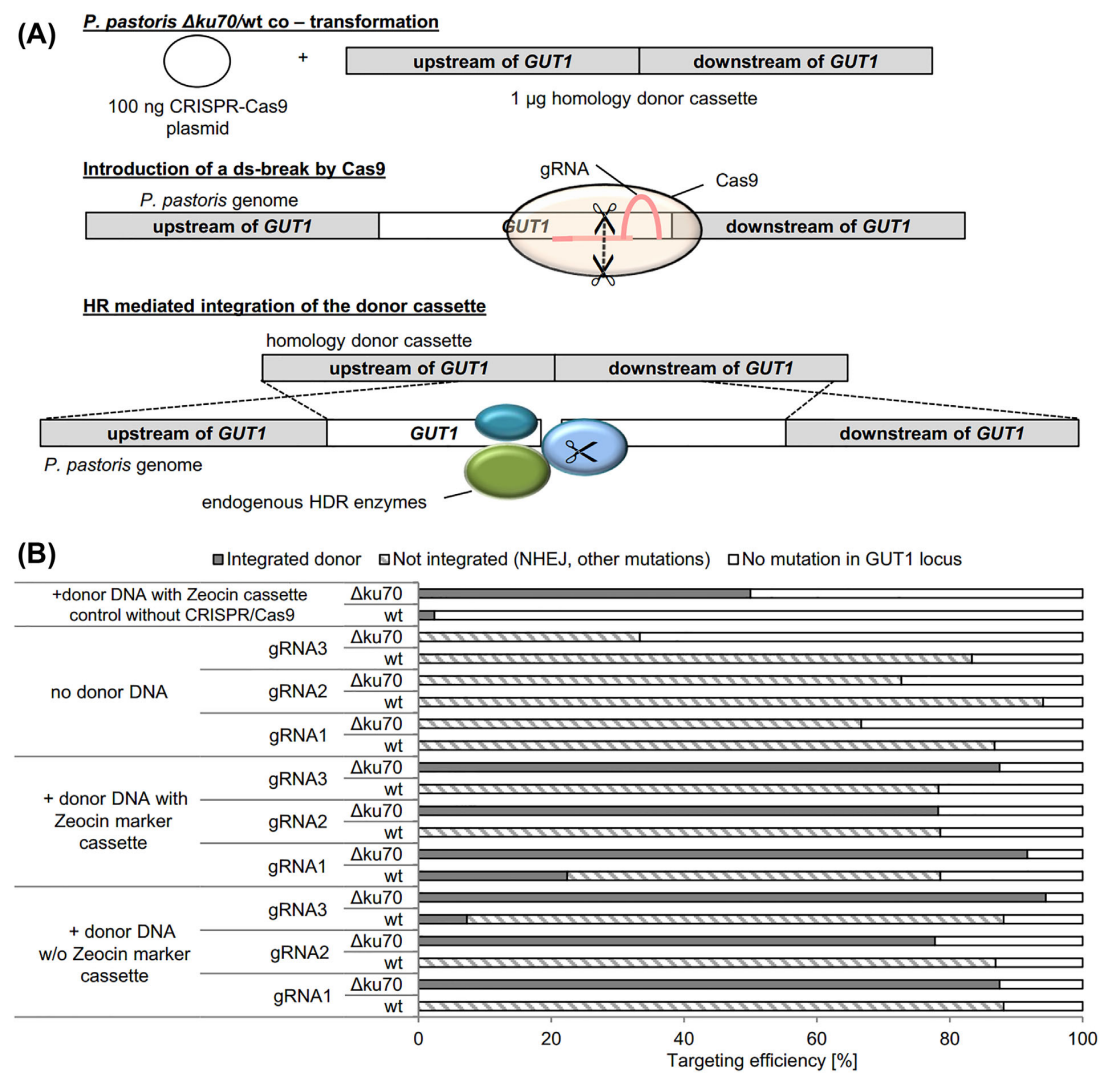
Markerless donor cassette integration with efficiencies approaching 100% in a *P. pastoris ku70* deletion strain. A, Principle of CRISPR/Cas9 mediated integration of donor cassettes to allow the complete deletion of genomic DNA. As first step *P. pastoris* Δ*ku70* was co‐transformed with 100 ng CRISPR/Cas9 plasmid and a linear homologous donor cassette (1 μg). The nuclease Cas9 introduced a double strand break at the target locus, which was the starting point for cellular repair. Host cell HR enzymes mediate the integration of the homologous donor fragment. CRISPR/Cas9 plasmids can be cured by growth on non‐selective media. The elements are not drawn to scale. B, CRISPR/Cas9 mediated integration of homologous donor cassettes does not strongly affect the wildtype strain, but improves specific integration in the *P. pastoris* Δ*ku70* strain. Competent cells were co‐transformed with 100 ng CRISPR/Cas9 circular plasmid DNA and 1 μg donor DNA to target *GUT1*. *P. pastoris* CBS 7435 wildtype results have previously been reported.^32^ As control solely one μg of the donor DNA was used for the transformation. Upon cultivating random transformants in 96‐DWPs, cell material was transferred with a metallic stamp on minimal media agar plates with either glucose (BMD1) or glycerol (BMG1) as carbon source to determine the targeting efficiency. The integration of the donor cassettes was verified by sequencing of >10 glycerol deficient transformants per CRISPR/Cas9 plasmid—donor DNA combination. Mean values and standard deviations of biological triplicates are shown

As next step *P. pastori*s Δ*ku70* and the wildtype strain were co‐transformed with CRISPR/Cas9 plasmids and marker‐free donor cassettes and selected for the presence of the episomal CRISPR/Cas9 plasmid: A *GUT1* donor cassette (previously reported^32^ consisting of two homologous arms directly fused together (total length 2 kbp, 1 μg used for transformation)) and an episomal CRISPR/Cas9 plasmid (100 ng) to target the *GUT1* CDS were co‐transformed (Figure 2A). Donor cassettes without a resistance marker allow to completely delete an ORF, to replace it with a different sequence or to insert sequences coding, for example, for a tag. The CRISPR/Cas9 plasmid can be simply removed by ARS plasmid loss by growing the strains on non‐selective media.^32^ By applying Zeocin selection, only transformants, which contained a CRISPR/Cas9 plasmid, were able to grow on selective agar plates. Ideally the homologous recombination rate of the donor would be increased up to 100% by the site specific strand break, which would allow the use of marker‐free homologous recombination cassettes for the replacement of genomic DNA. This turned out to be not the case in the wildtype strain, where NHEJ remained the preferred pathway for repair.^32^ However, high HR efficiencies for the marker‐free donor cassette of 78% (GUT1‐gRNA2) to 91% (GUT1‐gRNA1) were obtained with the *P. pastoris* Δ*ku70* strain (Figure 2B). Note that also the *P. pastoris* Δ*ku70* strain alone, without Cas9 and gRNA permits integration rates of a control cassette containing a resistance marker of approximately 50% at the *GUT1* locus (Figure 2B). Using Cas9 and the gRNA to target *GUT1* further improved the homologous recombination rate 1.6‐ to 1.9‐fold compared to the control (+donor DNA with Zeocin cassette, control without CRISPR/Cas9). Hence, the Cas9/gRNA system allowed to achieve marker less modifications even at a higher frequency than site specific marker integration in the Δ*ku70* strain.

We additionally used the control knockout cassette consisting of the Zeocin resistance cassette flanked by homology arms for CRISPR‐Cas9 co‐transformation experiments, in order to determine if the size of the donor DNA could have influenced the integration rates (2 kbp—donor DNA lacking the Zeocin marker cassette, 3.8 kbp—donor DNA bearing the Zeocin marker cassette). By applying Zeocin selection, it was not possible to discriminate transformants, which took up the CRISPR/Cas9 plasmid, the donor cassette or both recombinant DNA fragments. The HR efficiencies were in the same range as for the marker‐free donor cassettes: 78% for GUT1‐gRNA2 to 94% for GUT1‐gRNA3 suggesting that this length difference does not have a clear influence. For both donor fragments the lowest HR efficiency was obtained with GUT1‐gRNA2, indicating that this gRNA was generally less suited for such genome targeting approach.

The transformation rates of *P. pastoris* Δ*ku70* using the donor fragments and CRISPR/Cas9 plasmids were increased (between 200 and 300 CFUs/μg CRISPR/Cas9 plasmid DNA independently from the donor cassette and gRNA used) compared to transformations, where only a CRISPR/Cas9 plasmid had been transformed (less than 60 CFUs/μg CRISPR/Cas9 plasmid DNA), but were as expected still considerably lower than the wildtype strain (5000 CFUs/μg CRISPR/Cas9 plasmid DNA). The presence of the homology repair fragment seemed to reduce the toxicity of the CRISPR/Cas9 system in the *P. pastoris* Δ*ku70* strain.

This system provides for the first time the possibility to achieve marker less modifications in *P. pastoris* at frequencies approaching 100%. In a typical experiment of transforming 100 ng of CAS9/gRNA plasmid + donor, we obtained a few dozen colonies and nearly all of them were positive. Also in the case of *Y. lipolytica*, where the HR frequencies of donor cassettes were low to moderate in the wildtype strain (8% for the *TRP1* locus, 73% for the *MFE1* locus), integration frequencies of 100% could be obtained with a Δ*ku70* strain.^46^, ^47^

#### 3.1.2 Cas9 nickase to increase HR frequencies in *P. pastoris* wildtype was not successful

**Figure 3:**
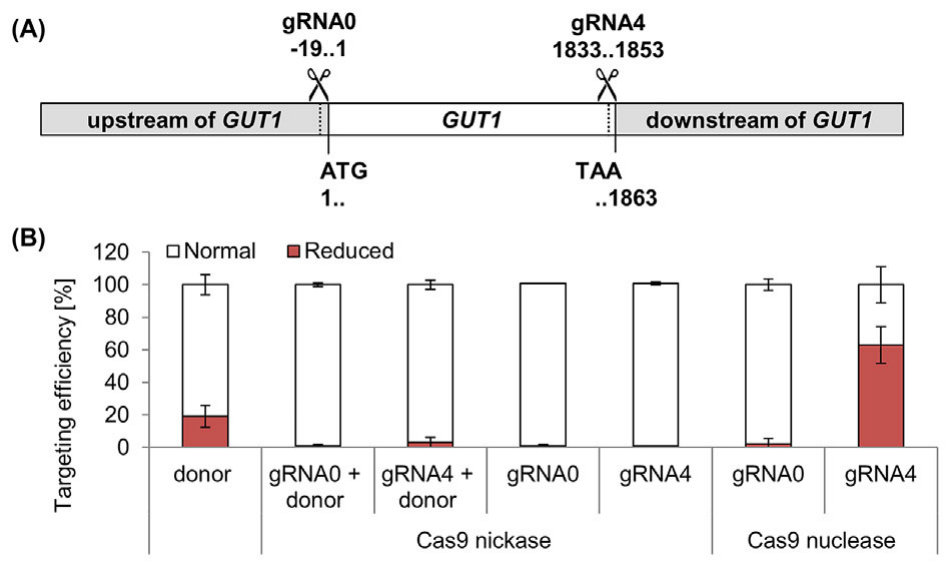
Use of the Cas9 nickase to increase HR frequencies in *P. pastoris* wildtype was not successful. A, GUT1‐gRNA0 and GUT1‐gRNA4 were designed, which should favor the formation of single stranded of homology arms for the integration of donor cassettes. B, *P. pastoris* CBS 7435 wildtype was transformed with CRISPR‐Cas9 plasmids bearing either *Hs*Cas9 or *Hs*Cas9 nickase and a gRNA to target *GUT1* (GUT1‐gRNA0 or GUT1‐gRNA4). Transformants were cultivated in 96‐DWPs and then stamped on minimal media plates containing glucose (BMD1) and glycerol (BMG1) as carbon source to determine the targeting efficiencies. The targeting efficiencies were only calculated by counting the glycerol deficient colonies and not by sequencing genomic DNA. Cas9 nickase transformants did not display a reduced growth phenotype compared to transformants with the Cas9 nuclease (*GUT1* deficient transformants obtained with GUT1‐gRNA4). In case of GUT1‐gRNA0/Cas9 nuclease either no cleavage had taken place due to a non‐functional gRNA or the cleavage did not result in a visible phenotype. Co‐transformations of the Cas9‐nickase plasmids (100 ng) and homology donor fragments (1 μg) yielded less *GUT1* deficient clones compared to transformations with solely the donor cassette

Although HR efficiencies could be increased in *P. pastoris* Δ*ku70* and we were able to integrate marker‐free donor cassettes, NHEJ remained the prevalent repair mechanism upon CRISPR/Cas9 targeted cleavage in *P. pastoris* wildtype strains. In order to increase HDR in *P. pastoris* wildtype strains, we constructed plasmids for the expression of the Cas9 nickase. This Cas9 variant creates single strand breaks due to the inactivation of one of its catalytic domains via the introduction of a point mutation.^34^, ^35^ The Cas9 nickase, which was cloned into *P. pastoris* vectors, bore the amino acid exchange D10 → A.^15^ By introducing single strand breaks, we intended to reduce the toxicity of the system and to mediate donor recombination, as demonstrated by Cong et al and Hsu et al.^36^, ^37^ We generated plasmids containing *CAS9* nickase and either GUT1‐gRNA0 or GUT1‐gRNA4 to target the *GUT1* locus. These gRNAs direct the cleavage in close proximity of the donor recombination sites (gRNA0 bp ‐19‐1 bp of *GUT1*, gRNA4 bp 1833‐1853 bp [numbering relative to start codon as +1]), which would benefit the formation of single stranded homology arms (Figure 3A) Targeting efficiencies (Δ*gut1* phenotype) of 2% were obtained for GUT1‐gRNA0 and of 64% for GUT1‐gRNA4 in combination with the wildtype Cas9 nuclease. The transformation of CRISPR/Cas9 plasmids bearing Cas9 nickase and either GUT1‐gRNA0 or GUT1‐gRNA4 did not result in glycerol deficient *P. pastoris* cells and also the co‐transformation of *Hs*Cas9 nickase plasmids and donor cassettes rendered most transformants unaffected, indicating that the single strand breaks were repaired correctly and the donor cassette had not been used as repair template (Figure 3B). *P. pastoris* Δ*ku70* was not transformed with Cas9 nickase plasmids, because single strand breaks are repaired independently from NHEJ,^26^, ^48^ and we did not expect differences compared to previous transformations.

#### 3.1.3 Autonomous replication of donor fragments increases HR rates in *P. pastoris* wildtype strains

**Figure 4:**
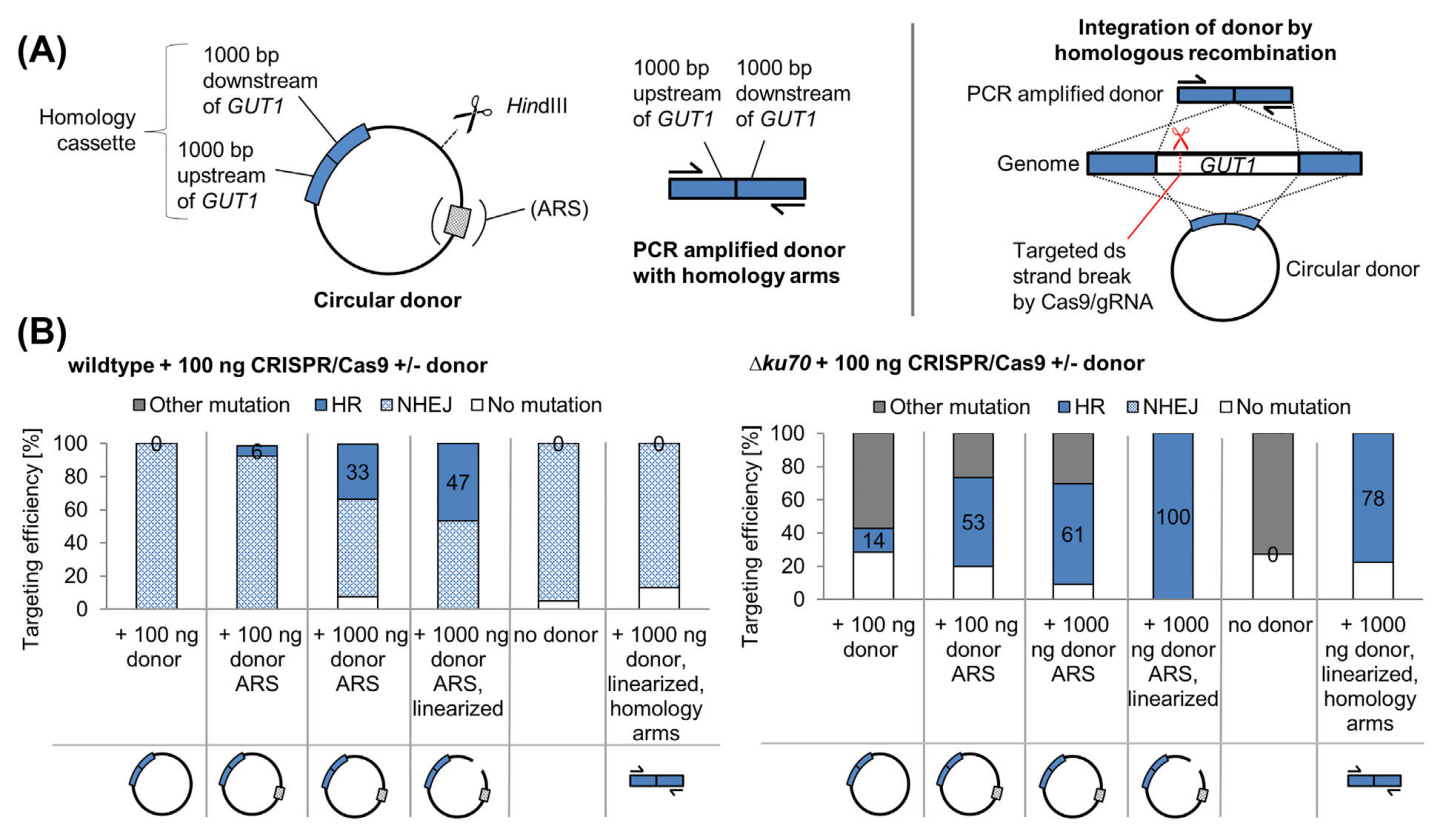
ARSs promote efficient CRISPR/Cas9 mediated integration of markerless donor fragments in the *P. pastoris* wildtype strain. A, Design of the donor cassettes (left side) and recombination events targeted (right side). A circular plasmid was used for the *P. pastoris* transformation, which contains 1000 bp regions 5′ upstream and 3′ downstream of *GUT1* (and an ARS for replication in yeast). As control the PCR amplified homology cassette consisting of the 1000 bp regions 5′ upstream and 3′ downstream of *GUT1* was used for the transformation. Due to sequence homology of the donor cassette the *GUT1* CDS can be removed by homologous recombination. B, *P. pastoris* wildtype (left side) and *Δku70* (right side) co‐transformation with 100 ng CRISPR‐Cas9 plasmid DNA (GUT1‐gRNA2) and various donor fragments: 100 ng circular donor DNA lacking ARS, 100 ng circular donor DNA with ARS, 1000 ng circular donor DNA with ARS, 1000 ng circular donor DNA with ARS linearized with *Hin*dIII, no donor DNA and 1000 ng PCR amplified donor DNA. Results on the PCR amplified donor cassette in the wildtype strain have previously been reported.^32^ Upon cultivating random transformants in 96‐DWPs, cell material was transferred with a metallic stamp on minimal media agar plates with either glucose (BMD1) or glycerol (BMG1) as carbon source to determine the targeting efficiency. The percentages of HR integrated donor fragments are denoted as numbers in the bar diagram. The integration of the donor cassettes was verified by sequencing of >10 glycerol deficient transformants per CRISPR/Cas9 plasmid—donor DNA combination. The ARS‐donor enables the integration of marker‐less donor fragments in almost 50% of the transformants, when 1000 μg linear donor DNA were used

As final attempt to increase HR frequencies for the integration of marker less donor cassettes in *P. pastoris* wildtype strains, we constructed donor cassettes bearing autonomous replication sequences (ARSs, Figure 4). Similar as observed for *Yarrowia lipolytica*,^46^ we assumed that the linear DNA used in the previous experiments (Figure 2) was not sufficiently sustained in the cells and that episomal maintenance might improve the HR frequency. Co‐transformations of CRISPR/Cas9 plasmids (100 ng) and various donor fragments were performed. Either 100 ng circular donor DNA lacking an ARS, 100 ng circular donor DNA containing an ARS, 1000 ng circular donor DNA with ARS, 1000 ng *Hin*dIII linearized donor DNA with ARS, no donor DNA or 1000 ng PCR amplified donor DNA (without ARS) were used for the transformations (Figure 4A). When co‐transforming a circular donor plasmid lacking an ARS, all *P. pastoris* wildtype transformants bore NHEJ mutations, similar to the transformations, where the donor was omitted. Co‐transforming 100 ng circular donor DNA containing an ARS yielded 92% clones with NHEJ mutations and a small proportion (6%), which integrated the donor fragment. When 1000 ng of the circular donor DNA containing an ARS were used, in 33% of the transformants the deletion of the *GUT1* CDS was observed by the integration of the homology donor. The HR frequencies could be further increased using the linearized donor DNA fragments containing the ARS (47% HR transformants, Figure 4B).

Interestingly, this experiment provided also insights on the recombination process in *P. pastoris*: The linear ends of the PCR amplified donor cassettes used as control are directly homologous to the genome (Figure 4A). In contrast, the ends of the linearized circular plasmid are different from the *GUT1* locus targeted, and the *GUT1* homologous sequences are placed relatively far (280 and 2917 bp) from the *Hin*dIII linearization site. Nonetheless, 47% of the constructs were integrated correctly. These results suggest, that the HR mechanism in *P. pastoris* “searches” for complementary sequences to the DSB introduced by Cas9/gRNA in the genome. Even more interestingly, also the circular plasmid yielded 33% correct integration. In this case, no linear ends are provided on the plasmid and the recombination event must be initiated by the DSB in the genome. Transformation efficiencies of ARS containing donors appeared to be slightly reduced. While the co‐transformation of 100 ng circular donor without ARS yielded ∼5000 cfu/μg CRISPR/Cas9 plasmid DNA, the transformation efficiency decreased to ∼1000 cfu/μg CRISPR/Cas9 plasmid DNA with 100 ng of circular donor containing an ARS. In case of the linearized ARS containing donor, the transformation efficiency decreased slightly more to ∼500 cfu/μg CRISPR/Cas9 plasmid DNA. Larger amounts of DNA in the transformation (and depending on the form circular/linear) hence appear to influence transformation rates in *P. pastoris*. Yet, the applicability of ARS containing donor fragments for genome engineering is not impaired, as the transformation rates are still sufficient to obtain many correct transformants (as discussed similarly for the Δ*ku70* strain without ARS cassettes).

To our knowledge, this is the first work showing the integration of marker‐less donor cassettes at reasonable rates in *P. pastoris* wildtype strains. These kinds of ARS donor cassettes are especially valuable for the replacement or removal of DNA sequences, where artifacts such as scars generated by site specific recombinases or selection marker cassettes might influence their functionality (eg, regulatory elements in promoters or “tagged” coding sequences). ARSs might be also placed on classical knockout cassettes to guarantee the maintenance of the cassette throughout the cell cycle, especially in the S/G2 phase were HR is predominant.^49^

When co‐transforming the NHEJ deficient Δ*ku70* strain with the CRISPR/Cas9 plasmid and 100 ng circular donor DNA lacking an ARS, 14% of the transformants integrated the donor fragment, which resulted in the deletion of the *GUT1* CDS (Figure 4B). The co‐transformation of *P. pastoris* Δ*ku70* with the CRISPR/Cas9 plasmid and 100 ng circular donor DNA containing an ARS yielded 53% HR transformants, 1000 ng circular donor DNA with ARS 61% HR transformants, and 1000 ng linearized donor DNA with ARS rendered all transformants glycerol deficient. However, in contrast to the *P. pastoris* wildtype strain, the PCR amplified donor bearing homology arms was also integrated at a high rate (78%), similar to transformations with the ARS donor cassettes. Also transformation rates with ARS containing donor fragments remained similarly low in the Δ*ku70* strain (50‐100 CFUs/μg CRISPR/Cas9 plasmid DNA) as when transforming regular PCR products (section 3.1.1). Hence while providing a major advantage in the wildtype strain, using ARS on the donor did not lead to clear improvements in the Δ*ku70* strain.

#### 3.1.4 Reduction of toxicity of the CRISPR/Cas9 system in *P. pastoris* using hf‐Cas9 was not successful

In order to increase the transformation efficiency and reduce the toxicity of the CRISPR/Cas9 system, we tried to implement a high fidelity Cas9 variant in *P. pastoris*. High fidelity Cas9 nucleases were generated by structure‐guided protein engineering and reduce off‐target effects, while maintaining robust on‐target cleavage.^18^, ^19^ Wildtype Cas9 can cleave off‐target sites that are not fully complementary to the gRNA, when the binding strength to the non‐complementary DNA strand is high. If interactions between Cas9 and the non‐complementary DNA strand are reduced due to amino acid exchanges, an increase in the specificity had been observed. Four amino acids in the Cas9 sequence were exchanged according to Kleinstiver et al^18^ to prepare the off‐target free version of Cas9 (497N → A, 661R → A, 695Q → A, 926Q → A), hf‐Cas9. The hf‐Cas9 coding sequence was cloned into the vector previously used (bearing GUT1‐gRNA1 flanked by ribozymes under the control of the bidirectional *HTX1* promoter). The targeting efficiency for the *GUT1* locus was similar (80%), compared to the transformations using unmutated, wildtype Cas9 (91%), indicating that on‐target activities had been retained (data not shown). The indel mutations in the *GUT1* locus introduced by either Cas9 or hf‐Cas9 were verified by sequencing. In two clones bearing Cas9, a single bp (G‐C) located four bps upstream of the protospacer adjacent motif (PAM, for Cas9: AGG) was deleted (CGAGTACTCTACCTCTCTCAGG, 2/2 sequenced), as well as in one of the sequenced clones expressing hf‐*HsCAS9* (1/2 sequenced). In the second hf‐*Hs*Cas9 transformant, one bp located 3 bp upstream PAM was deleted (CGAGTACTCTACCTCTGTCAGG). The PAM is a 3 bp DNA sequence immediately following the Cas9 nuclease target DNA sequence. PAM is essential for Cas9 mediated DNA unwinding and subsequent DNA cleavage.^50^ These initial implementation studies were performed in a *P. pastoris* wildtype strain. As next step we transformed *P. pastoris* Δ*ku70* where transformation efficiencies were 10‐fold reduced due to the impaired NHEJ‐repair pathway (Figure 1). Possible off‐target cleavage is an additional burden to the CRISPR/Cas9 transformants. Thus we expected an increase in the transformation efficiency, if solely on‐target cleavage had taken place (and was a major factor affecting the CRISPR/Cas9 efficiency in *P. pastoris*). The transformations of *P. pastoris* wildtype cells with either hf‐Cas9 or Cas9 and GUT1‐gRNA1 yielded 5000 CFUs/μg DNA. Similarly, when transforming *P. pastoris* Δ*ku70*, transformation efficiencies of 20‐40 CFUs/μg DNA were obtained with either hf‐Cas9 or Cas9 and GUT1‐gRNA1. Additional co‐transformations with homology donor cassettes yielded similar transformation efficiencies with Cas9 and hf‐Cas9 (20‐60 CFUs/μg CRISPR‐Cas9 plasmid DNA). Thus inferred from this data, hf‐Cas9, generated in this work, appears to show similar on‐ and off‐target activities compared to the parental Cas9 enzyme in our experimental setup in *P. pastoris*. The use of hf‐Cas9 does not appear to provide advantages over the wildtype Cas9 enzyme in *P. pastoris*.

### 3.2 DNA deletion by multiplexing

**Figure 5:**
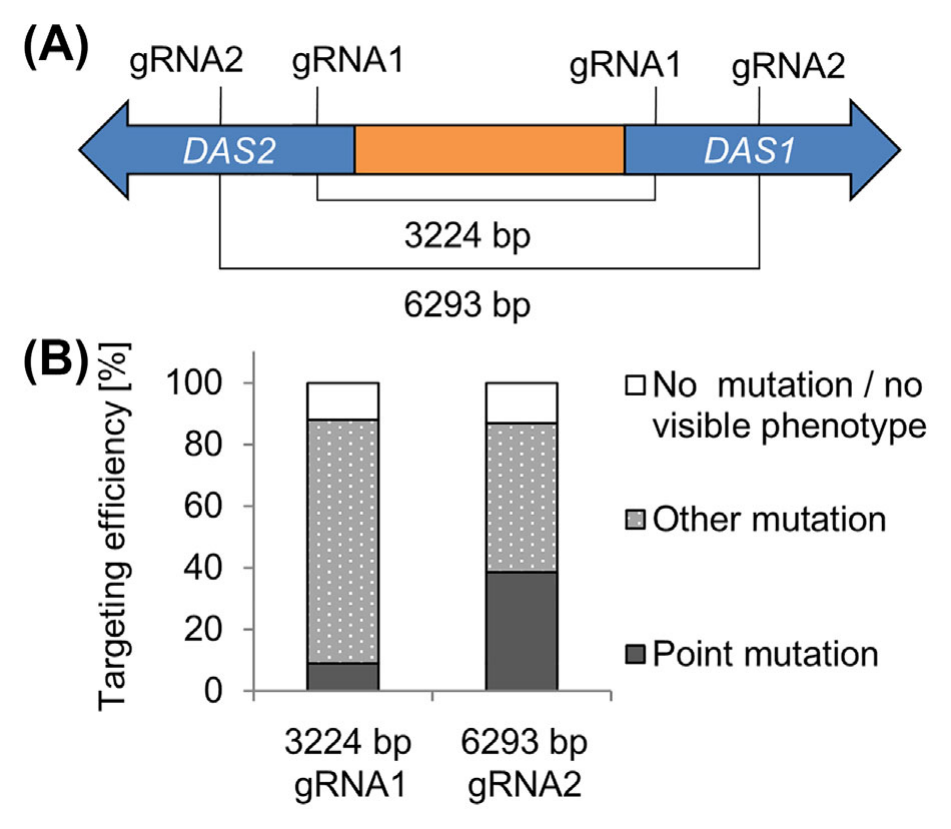
Dual cutting gRNAs for DNA removal. A, The *P. pastoris DAS1/DAS2* locus consisting of *DAS1* and *DAS2*, which are located in the reverse complement orientation and are interspaced with the respective promoters and *HOB3*, encoding a putative guanosine nucleotide exchange factor. Due to 91% sequence identity a single gRNA can be designed to introduce double strand breaks in the *DAS1* and *DAS2* gene. B, *P. pastoris* CBS 7435 wildtype was transformed with CRISPR‐Cas9 plasmids bearing *Hs*Cas9 and either *DAS1*/*DAS2*‐gRNA1 or *DAS1*/*DAS2*‐gRNA2. Transformants were cultivated in 96‐DWPs and then stamped on minimal media plates containing glucose (BMD1) and methanol (BMM1) as carbon source to determine the targeting efficiencies. The type of mutation was elucidated by sequencing of >10 methanol deficient transformants/construct. Mean values and standard deviations of biological triplicates are shown. Increasing the distance of the targeting sites increases the frequencies of NHEJ mutations and reduces the probability for sequence removal

Besides gene replacement by the co‐transformation of donor fragments, DNA removal was investigated using gRNAs, which direct multiple Cas9 cleavages in close proximity in the *P. pastoris* genome. Thereby sequences from the genome could be removed without requiring a donor cassette to repair the gap (Figure 5). The *DAS1/DAS2* locus was selected for the implementation. In *P. pastoris* two isoforms of the dihydroxyacetone synthase, *DAS1* and *DAS2*, occur, which are 91% identical.^51^, ^52^, ^53^ These genes are located in the reverse complement orientation and are interspaced with the respective promoters and *HOB3*, encoding a putative guanosine nucleotide exchange factor. *P. pastoris DAS1* and *DAS2* double knockout strains display a reduced growth on methanol similar to *AOX1* deficient mut^S^ strains. Whereas single knockouts have a similar growth rate on methanol as sole carbon source, compared to *P. pastoris* wildtype strains.^53^ Due to the high sequence identity a single gRNA can be designed, which directs cleavage in both, the *DAS1* and the *DAS2* gene (Figure 5).

Two different gRNAs (*DAS1*/*DAS2*‐gRNA1 and *DAS1*/*DAS2*‐gRNA2) were cloned in CRISPR/Cas9 plasmids bearing *Hs*Cas9. The *DAS1*/*DAS2*‐gRNA1 cleavage sites were interspaced by a 3224 bp fragment, whereas the *DAS1*/*DAS2*‐gRNA2 cleavage sites were interspaced by a 6293 bp fragment (Figure 5A). The overall targeting efficiencies for the simultaneous cleavage at the *DAS1* and *DAS2* locus were similar for both gRNAs (87% for *DAS1*/*DAS2*‐gRNA1 and 86% for *DAS1*/*DAS2*‐gRNA2). However the type of mutation differed depending on the distance of cleavage sites (Figure 5B). Shorter distances led to the removal of DNA fragments, however, chromosomal rearrangements and translocations were observed rather than clean cuts. PCR amplifying and sequencing of these transformants resulted either in no PCR product or sequencing reads, which could only be aligned partially. Local translocations might be favored due to the palindromic nature of the target sequences. *DAS1*/*DAS2*‐gRNA1 mediated cleavage appeared to predominantly trigger rearrangement/translocation events at the *DAS1*/*DAS2* locus (79%). When using *DAS1*/*DAS2*‐gRNA2 48% of the transformants bore rearrangement/translocation mutations, and the number of transformants containing point mutations at the Cas9 cleavage site increased to 38.6% compared to *DAS1*/*DAS2*‐gRNA1 transformants (8.8%).

### 3.3 CRISPR/Cas9 expression from geneticin marker plasmids

*P. pastoris* production strains often contain a Zeocin cassette integrated in the genome, which was required for the initial selection for the gene of interest. At later protein production stages the Zeocin resistance is not required anymore, since the genomic integration of expression cassettes results in stable expression strains, which maintain the integrated cassette in the absence of selective pressure. However re‐transformations of these strains with plasmids containing the Zeocin resistance marker, for example, for the expression of helper genes/chaperones and also CRISPR/Cas9 mediated strain engineering with the plasmids currently established^32^ cannot be performed.

**Figure 6:**
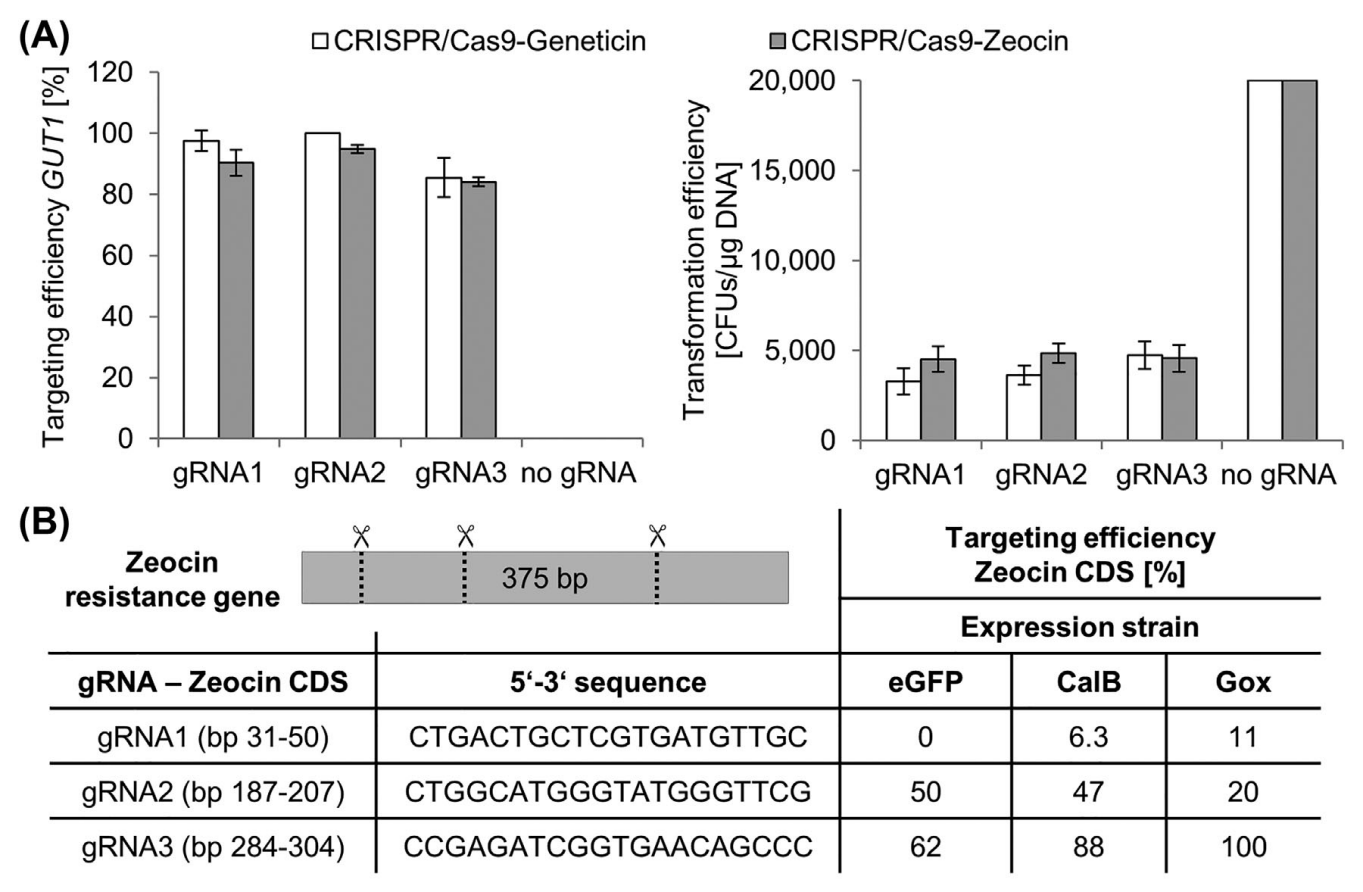
Characterization of the CRISPR/Cas9‐Geneticin system for *P. pastoris* and application for marker recycling of Zeocin resistance cassette containing strains. A, *P. pastoris* cells were transformed with CRISPR/Cas9 plasmids containing gRNAs to target *GUT1* and a Geneticin resistance marker cassette. The transformants were cultivated in 96‐DWPs and stamped on minimal media plates with different carbon sources (glucose [BMD1] and glycerol [BMG1]) to determine the targeting efficiency. The Geneticin results are compared to the CRISPR/Cas9‐Zeocin system published previously.^32^ Mean values and standard deviation of biological triplicates are shown. In case of the Geneticin GUT1‐gRNA2, all replicates yielded 100% correct integration, hence no standard deviation is shown. B, The CRISPR/Cas9‐Geneticin plasmid was used for marker recycling in existing *P. pastoris* production strains. Several other *P. pastoris* strains bearing a Zeocin resistance cassette and a reporter gene/enzyme expression cassette (eGFP, CalB, Gox) integrated in the genome were transformed with CRISPR/Cas9 plasmids harboring gRNAs to target the Zeocin resistance gene of the pPpT4 vector.^23^ Indel mutations were introduced in the CDS of the Zeocin resistance gene, which rendered the strains Zeocin sensitive. The targeting efficiencies were determined by stamping the randomly picked transformants on YPD and YPD‐Zeocin (50 μg/mL) agar plates. Mean values and standard deviations of biological triplicates are shown

In order to expand the CRISPR/Cas9 toolset for *P. pastoris* we generated a CRISPR/Cas9‐Geneticin plasmid bearing a Geneticin resistance cassette, which can be used to introduce targeted mutations in preexisting Zeocin resistant *P. pastoris* strains. Moreover we also applied this plasmid for marker recycling^54^ by transforming Zeocin resistant *P. pastoris* strains with CRISPR/Cas9‐Geneticin plasmids containing gRNAs to target the Zeocin resistance gene (Figure 6). Using this approach Zeocin sensitive *P. pastoris* strains were generated, which can be re‐transformed with plasmids bearing Zeocin resistance markers.

As first step, the CRISPR/Cas9‐Geneticin system was compared to the already published CRISPR/Cas9‐Zeocin vectors by targeting the *GUT1* locus (Figure 6A). The *CAS9*/gRNA expression cassette of the newly generated CRISPR/Cas9‐Geneticin plasmid were identical to the Zeocin based designs used previously^32^ and in this study. Only the resistance marker cassette was exchanged. We transformed a *P. pastoris* wildtype strain with CRISPR/Cas9 plasmids lacking the gRNAs, and harboring either the Zeocin resistance marker or the Geneticin resistance marker. Using the CRISPR/Cas9‐Zeocin and the CRISPR/Cas9‐Geneticin plasmid more than 20 000 CFUs/μg DNA were obtained. However, it may be noted that in previous experiments a reduced number of CRISPR/Cas9‐Geneticin transformants had been observed (data not shown). Transformation efficiencies of 3280 to 4735 CFUs/μg DNA were obtained with the CRISPR/Cas9‐Geneticin plasmids containing gRNAs. Using these plasmids targeting efficiencies of 85‐100% for the *GUT1* locus similar to the transformations of the CRISPR/Cas9‐Zeocin plasmids (87‐94% for *GUT1* and different gRNAs,^32^) were obtained (Figure 6A). Thus the described CRISPR/Cas9 experiments with *P. pastoris* can be performed with Gen/KanMX based vectors as well as with vectors using the Zeocin resistance gene.

For the marker recycling and the for inactivating the resistance marker gene in existing Zeocin based *P. pastoris* strains, different gRNAs were designed (ZEO‐gRNA1, ZEO‐gRNA2, ZEO‐gRNA3) to introduce indels in the CDS of the Zeocin resistance gene and to inactivate the protein functionality (Figure 6B). *P. pastoris* strains harboring plasmids for the expression of either enhanced green fluorescent protein (eGFP), *C. antarctica* lipase B (CalB) or glucose oxidase (Gox) with Zeocin resistance markers were transformed with CRISPR/Cas9‐Geneticin plasmids to target the CDS of the Zeocin resistance gene. The targeting efficiencies varied depending on the gRNA used: ZEO‐gRNA1 yielded the lowest targeting efficiencies in the three *P. pastoris* strains (0‐11%). Moderate targeting efficiencies were obtained with ZEO‐gRNA2 (20‐50%), whereas ZEO‐gRNA3 enabled highly efficient gene targeting (62‐100%) (Figure 6B). CRISPR/Cas9 plasmid curing was performed by growing the strains on non‐selective media. This strategy can be adapted for the recycling of various dominant selection markers including antibiotics and auxotrophy markers, and offers an alternative to the existing recombinase systems (Flippase/FRT, Cre/loxP, reviewed by Vogl et al^54^), where the recognition sites have to be incorporated in the plasmid prior to the transformation.^12^ In addition, also two gRNAs could be used to completely excise the marker gene, as discussed for the *DAS1/2* locus (section 3.2 and Figure 3). However, note that the coding sequence of the Zeocin resistance gene of the pPpT4 plasmids family,^23^ that was used as the basis in our work, is different from other commonly used vectors (eg, Invitrogen vectors). Hence the gRNAs reported here for Zeocin marker recycling would possibly need to be adapted for the use with other vector families.

## 4 CONCLUSION AND OUTLOOK

Continuous developments of CRISPR/Cas9 systems broaden the range of applications to engineer expression hosts including the methylotrophic yeast *P. pastoris*. In this work, efficient and precise CRISPR/Cas9 induced HR was on the one hand demonstrated by transforming the NHEJ‐impeded *P. pastoris* Δ*ku70* strain and on the other hand also efficiencies for the *P. pastoris* wildtype strain were improved by using donor cassettes with ARSs. Thereby also homology fragments lacking selection marker cassettes were correctly integrated at high frequencies. In case of the *P. pastoris* Δ*ku70* strain low transformation rates were obtained. However, in a typical experiment of transforming 100 ng CRISRP/Cas9 plasmid DNA and 1 μg linear donor DNA resulted in a few dozen colonies and nearly all of them were positive. Inhibiting NHEJ to increase the HR efficiency in combination with CRISPR/Cas9 gene targeting had been successfully applied in alternative yeast species,^28^, ^46^, ^47^ mammalian cell lines, and mice^55^ before and is now for the first time applicable for *P. pastoris* Δ*ku70* strains. Furthermore, for the integration of homology donor fragments in a *P. pastoris* wildtype strain the addition of ARSs increased the integration rates from as little as 0% up to approximately 50%. To our knowledge this is the first report demonstrating the integration of markerless donor cassettes at reasonable rates in *P. pastoris* wildtype strain, which enables novel engineering strategies. Markerless donor cassettes are especially valuable for the replacement or removal of DNA sequences, where artifacts such as scars generated by site specific recombinases or selection marker cassettes might influence their functionality (eg, regulatory elements in promoters or “tagged” coding sequences). ARSs might be also placed on classical knockout cassettes to guarantee the maintenance of the cassette throughout the cell circle, especially in the S/G2 phase were HR is predominant and thereby favor their integration.^49^ Hence with these systems, either close to 100% correct integration can be achieved using the Δ*ku70* strain (at the expense of total transformation efficiency) or the wildtype strain can be used allowing ∼50% correct integration. To expand the CRISPR/Cas9 toolset of *P. pastoris* we also generated CRISPR‐Cas9 plasmids, where transformants can be selected with Geneticin. Those can also be applied to introduce targeted mutations in Zeocin resistant *P. pastoris* strains. Moreover CRISPR/Cas9 plasmids with different selection markers enable marker recycling by using alternating gRNAs to delete resistance marker genes. The results presented here may aid to make the CRISPR/Cas9 technology even more appealing to the *P. pastoris* community, provide a deepened understanding of CRISPR/Cas9 for strain engineering and might be adapted for alternative production hosts. For the first time also to the introduction of scarless tags, point mutations in genome, protein fusions or deletions of whole genome sequence stretches will be enabled employing these tools.

## Supporting information

Additional Supporting Information may be found online in the supporting information tab for this article.

Supporting Information S1.

Supporting Information S2.
