# Supplementary material for: Expanding the CRISPR/Cas9 toolkit for Pichia pastoris with efficient donor integration and alternative resistance markers
Source: J Cell Biochem. 2017 Dec 26;119(4):3183–98. doi: 10.1002/jcb.26474 (PMC5887973; doi:10.1002/jcb.26474)
Supplement: Supplementary file 2 — Supporting Information S2. [file JCB-119-3183-s002.pdf]

Supplementary materials

Expanding the CRISPR-Cas9 toolkit for *Pichia pastoris* with efficient donor integration and alternative resistance makers

S1: Primers and gBlocks 2
S2: Plasmids 5
S3: Sequencing of the *GUT1* locus upon CRISPR/Cas9 cleavage in *P. pastoris Δku70*. 6

S1: Primers and gBlocks

Primers and gBlocks used in this study are listed here. The generation of some plasmids has previously been described in: Weninger *et al.*, (2016) "Combinatorial optimization of CRISPR/Cas9 expression enables precision genome engineering in the methylotrophic yeast *Pichia pastoris*", Journal of Biotechnology, 235:139-149.

**Primer name**	**Sequence 5' --> 3'**
**Primers for the amplification of the *GUT1* locus**	
GUTout3prR1	ATGAAGTTAGTAAGGTTCTTGATGAAGC
PGUTseq2	GGTACTTTGCCGACTCCTC
5prGUT1_fwd	tgggttcaatggcgtttgagttag
GUT1-3-fw	GAAAAGGTTTACTATCCCGATTTAGGCGAAAAGAG
GUT1-438-seq-fw	Cttcagagagcttgccgtagaaagaacg
GUT1-525-seq-rv	Gttggaatcccagtaaggggcaaacaatcctg
**CRISPR/Cas9 nickase constructs**	
RZ-GUT1-gRNA0-RZ	CCAGTTCAAGTTACCTAAACAAATCAAATCTGTGCTGATGAGTCCGTGAGGACGAAACGAGTAAGCTCGTCcacagatatatctactataaGTTTTAGAGCTAGAAATAGCAAGTTAAAATAAGGCTAGTCCGTTATCAACTTGAAAAAGTGGCACCGAGTCGGTGCTTTTGGCCGGCATGGTCCCAGCCTCCTCGCTGGCGCCGGCTGGGCAACATGCTTCGGCATGGCGAATGGGACTCAAGAGGATGTCAGAATGCC
RZ-GUT1-gRNA4-RZ	CCAGTTCAAGTTACCTAAACAAATCAAAGCCTTTCTGATGAGTCCGTGAGGACGAAACGAGTAAGCTCGTCaaaggcaaagggctggcttaGTTTTAGAGCTAGAAATAGCAAGTTAAAATAAGGCTAGTCCGTTATCAACTTGAAAAAGTGGCACCGAGTCGGTGCTTTTGGCCGGCATGGTCCCAGCCTCCTCGCTGGCGCCGGCTGGGCAACATGCTTCGGCATGGCGAATGGGACTCAAGAGGATGTCAGAATGCC
DAS1TT-hsCas9-rv	TAAAACTGTAAAGACTTCCCGTCTCGAGTTAAACTTTTCTTTTCTTCTTTGGGTCTCCAC
HsCas9-D10A-fw	TATATTAAAACTACAACAGAATTCCGAAACGATGGACAAGAAGTACTCCATTGGGCTCGCTATCGGCACAAACAGCGTCG
**hf-HsCas9**	
P_AOX1_syn_pUCORI_Gibs_fv	CTTTTGCTGGCCTTTTGCTCACATGTTCTTTCCTGCGGTACCC
HsCas9_rev	CTCGATTCACGCATGAACACCAA
HsCas9_fv	CTTATCCACGACTTCCTCGAAGTTC
HDV_AOX1TT_Gibs_rev	CAAATGGCATTCTGACATCCTCTTGAGTCCCATTCGCCATGCCGAA

gBlock_hf-HsCas9	TTGGTGTTCATGCGTGAATCGAGAATTTGGGCCACGTGCTTGGTGATAGCGCGTGTCTCA<br>ACAAGCTGCCTTTTGATGAAGCCGGCTTTATCCAACTCAGACAGGCCACCTCGTTCAGCCT<br>TAGTCAGATTATCGAACTTCCGTTGTGTGATCAGTTTGGCGTTCAGCAGCTGCCGCCAATA<br>ATTTTTCATTTTCTTGACAACTTCTTCTGAGGGGACGTTATCACTCTTCCCTCTATTTTTATC<br>GGATCTTGTCAACACTTTATTATCAATAGAATCATCTTTGAGAAAAGACTGGGGCACGATAT<br>GATCCACGTCGTAGTCGGAGAGCCGATTGATGTCCAGTTCCTGATCCACGTACATGTCCC<br>TGCCGTTCTGCAGGTAGTACAGGTAGAGCTTCTCATTCTGAAGCTGGGTGTTTTCAACTGG<br>GTGTTCCTTAAGGATTTGGGACCCCAGTTCTTTTATACCCTCTTCAATCCTCTTCATCCTTT<br>CCCTACTGTTCTTCTGTCCCTTCTGGGTAGTTTGGTTCTCTCGGGCCATCTCGATAACGAT<br>ATTCTCGGGCTTATGCCTTCCCATTACTTTGACGAGTTCATCCACGACCTTAACGGTCTGC<br>AGTATTCCCTTTTTGATAGCTGGGCTACCTGCAAGATTAGCGATGTGCTCGTGAAGACTGT<br>CCCCCTGGCCAGAAACTTGTGCTTTCTGGATGTCCTCCTTAAAGGTGAGAGAGTCATCATG<br>GATCAAAGCCATGAAGTTCCGGTTGGCAAATCCATCGGACTTAAGAAAATCCAGGATTGTC<br>TTTCCACTCTGCTTGTCTCGGATCCCATTGATCAGTTTTCTTGACAGAGCCCCCCATCCTG<br>TATATCGGCGCCTCTTGAGCTGTTTCATGACTTTGTCGTCGAAGAGATGAGCGTAAGTTTT<br>CAAGCGTTCTTCAATCATCTCCCTATCTTCAAACAACGTAAGGGTGAGGACAATGTCCTCA<br>AGAATGTCCTCGTTCTCCTCATTGTCCAGGAAGTCCTTGTCTTTAATGATTTTCAGGAGATC<br>GTGATACGTTCCCAGGGATGCGTTGAAGCGATCCTCCACTCCGCTGATTTCAACAGAGTC<br>GAAACATTCAATCTTTTTGAAATAGTCTTCTTTGAGCTGTTTCACGGTAACTTTCCGGTTCG<br>TCTTGAAGAGGAGGTCCACGATAGCTTTCTTCTGCTCTCCAGACAGGAATGCTGGCTTTCT<br>CATCCCTTCTGTGACGTATTTGACCTTGGTGAGCTCGTTATAAACTGTGAAGTACTCGTAC<br>AGCAGAGAGTGTTTAGGAAGCACCTTTTCGTTAGGCAGATTTTTATCAAAAGCAGTCATCC<br>TTTCGATGAAGGACTGGGCAGAGGCCCCCTTATCCACGACTTCCTCGAAGTTC
3UTRGUTR	GTGTTTGCTGTAGGATGACCTAGATTTAAATATAAGAGGAAACAACGTTCGTATCGTGA
seq-pGUT1-332..308-fwd	tgggttcaatggcgtttgagttag
**Episomal donor cassettes**	
ARG4TT-ARS-fw	TCGTCTCCAGCAGCATCCTCGCATTGGTACTCGAGATAAGCTGGGGGAACATT
pUORI-ARS-rv	CTACGGGGTCTGACGCTCAGTG TCGACAATTAATATTTACTTATTTTGGTCAAC
Pjet-ARS-fw	AATAGGCGTATCACGAGGCCGCCCCTGCATCGAGATAAGCTGGGGGAACATT
Pjet-ARS-rv	TTATTTGGCAAAAATAATATAATTCGGCTCGACAATTAATATTTACTTATTTTGGTCAAC
**CRISPR/Cas9 constructs for DNA deletion at the DAS1/DAS2 locus**	
RZ-DAS1/2-gRNA1-RZ	CCAGTTCAAGTTACCTAAACAAATCAAAACCTCACTGATGAGTCCGTGAGGACGAAACGAG<br>TAAGCTCGTCTGAGGTTACCACTGGTCCCCGTTTTAGAGCTAGAAATAGCAAGTTAAAATA<br>AGGCTAGTCCGTTATCAACTTGAAAAAGTGGCACCGAGTCGGTGCTTTTGGCCGGCATGG<br>TCCCAGCCTCCTCGCTGGCGCCGGCTGGGCAACATGCTTCGGCATGGCGAATGGGACTC<br>AAGAGGATGTCAGAATGCC
RZ-DAS1/2-gRNA2-RZ	CCAGTTCAAGTTACCTAAACAAATCAAACTCAACCTGATGAGTCCGTGAGGACGAAACGAG<br>TAAGCTCGTCGTTGAGGCCTATGTCGCATAGTTTTAGAGCTAGAAATAGCAAGTTAAAATA<br>AGGCTAGTCCGTTATCAACTTGAAAAAGTGGCACCGAGTCGGTGCTTTTGGCCGGCATGG<br>TCCCAGCCTCCTCGCTGGCGCCGGCTGGGCAACATGCTTCGGCATGGCGAATGGGACTC<br>AAGAGGATGTCAGAATGCC
Das2_rev	GTCCTACAGGTAAACAGATAGCCACTACG
Das1_fw	CATCAGATATTATCATCGCGGCTTACG
Das2_fw	CGATACTTCTCCACATTCAGTCATAGATGG
Das1_rev	CCTCAGGTCTCGGTTAGCCTCTAGG
**CRISPR/Cas9 plasmid bearing the Geneticin resistance cassette**	
RZ-GUT1-gRNA1-RZ	CCAGTTCAAGTTACCTAAACAAATCAAATACTCGCTGATGAGTCCGTGAGGACGAAACGAG<br>TAAGCTCGTCCGAGTACTCTACCTCTGCTCGTTTTAGAGCTAGAAATAGCAAGTTAAAATAA<br>GGCTAGTCCGTTATCAACTTGAAAAAGTGGCACCGAGTCGGTGCTTTTGGCCGGCATGGT<br>CCCAGCCTCCTCGCTGGCGCCGGCTGGGCAACATGCTTCGGCATGGCGAATGGGACTCA<br>AGAGGATGTCAGAATGCC
RZ-GUT1-gRNA2-RZ	CCAGTTCAAGTTACCTAAACAAATCAAAATTGCACTGATGAGTCCGTGAGGACGAAACGAG<br>TAAGCTCGTCTGCAATTTCCTCAGCCAGGCGTTTTAGAGCTAGAAATAGCAAGTTAAAATA<br>AGGCTAGTCCGTTATCAACTTGAAAAAGTGGCACCGAGTCGGTGCTTTTGGCCGGCATGG<br>TCCCAGCCTCCTCGCTGGCGCCGGCTGGGCAACATGCTTCGGCATGGCGAATGGGACTC<br>AAGAGGATGTCAGAATGCC

RZ-GUT1-gRNA3-RZ	CCAGTTCAAGTTACCTAAACAAATCAAAAACAACCTGATGAGTCCGTGAGGACGAAACGAG TAAGCTCGTCGTTGTTTGGTCCAAGAAGACGTTTTAGAGCTAGAAATAGCAAGTTAAAATA AGGCTAGTCCGTTATCAACTTGAAAAAGTGGCACCGAGTCGGTGCTTTTGGCCGGCATGG TCCCAGCCTCCTCGCTGGCGCCGGCTGGGCAACATGCTTCGGCATGGCGAATGGGACTC AAGAGGATGTCAGAATGCC
Zeocin resistance gene (CDS)	ATGGCTAAACTCACCTCTGCTGTTCCAGTC**CTGACTGCTCGTGATGTTGC**TGGTGCTGTTG AGTTCTGGACTGATAGACTCGGTTTCTCCCGTGACTTCGTAGAGGACGACTTTGCCGGTG TTGTACGTGACGACGTTACCCTGTTCATCTCCGCAGTTCAGGACCAGGTTGTGCCAGACA ACACT**CTGGCATGGGTATGGGTTCG**TGGTCTGGACGAACTGTACGCTGAGTGGTCTGAG GTCGTGTCTACCAACTTCCGTGATGCATCTGGTCCAGCTATGA**CCGAGATCGGTGAACAG CCC**TGGGGTCGTGAGTTTGCACTGCGTGATCCAGCTGGTAACTGCGTGCATTTCGTCGCA GAAGAGCAGGACTAA
ZEO-gRNA1	CCAGTTCAAGTTACCTAAACAAATCAAA**agtcag**CTGATGAGTCCGTGAGGACGAAACGAG TAAGCTCGTC**CTGACTGCTCGTGATGTTGC**GTTTTAGAGCTAGAAATAGCAAGTTAAAATA AGGCTAGTCCGTTATCAACTTGAAAAAGTGGCACCGAGTCGGTGCTTTTGGCCGGCATGG TCCCAGCCTCCTCGCTGGCGCCGGCTGGGCAACATGCTTCGGCATGGCGAATGGGACTC AAGAGGATGTCAGAATGCC
ZEO-gRNA2	CCAGTTCAAGTTACCTAAACAAATCAAA**tgccag**CTGATGAGTCCGTGAGGACGAAACGAG TAAGCTCGTC**CTGGCATGGGTATGGGTTCG**GTTTTAGAGCTAGAAATAGCAAGTTAAAATA AGGCTAGTCCGTTATCAACTTGAAAAAGTGGCACCGAGTCGGTGCTTTTGGCCGGCATGG TCCCAGCCTCCTCGCTGGCGCCGGCTGGGCAACATGCTTCGGCATGGCGAATGGGACTC AAGAGGATGTCAGAATGCC
ZEO-gRNA3	CCAGTTCAAGTTACCTAAACAAATCAAA**tctcgg**CTGATGAGTCCGTGAGGACGAAACGAG TAAGCTCGTC**CCGAGATCGGTGAACAGCCC**GTTTTAGAGCTAGAAATAGCAAGTTAAAAT AAGGCTAGTCCGTTATCAACTTGAAAAAGTGGCACCGAGTCGGTGCTTTTGGCCGGCATG GTCCCAGCCTCCTCGCTGGCGCCGGCTGGGCAACATGCTTCGGCATGGCGAATGGGACT CAAGAGGATGTCAGAATGCC

S2: Plasmids

Sequence files of the following plasmids are provided in genbank (.gb) format.

pPpT4_pHTX1-hf-HsCas9-GUT1-gRNA1

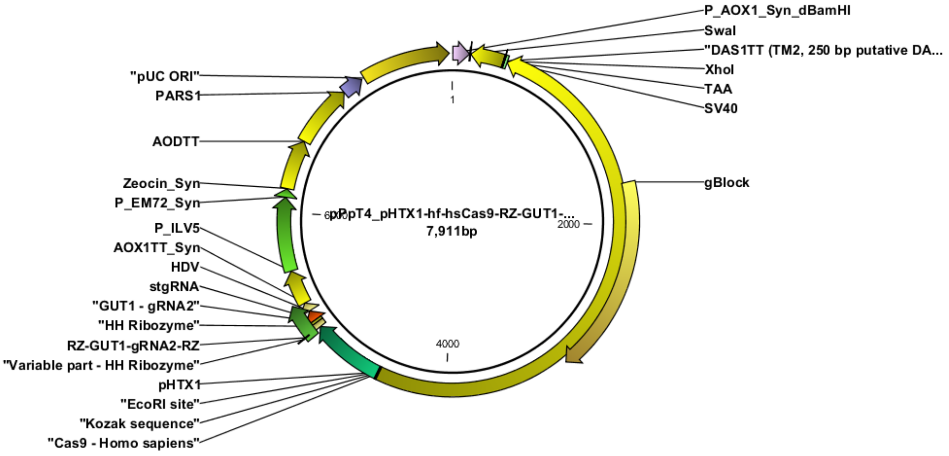

pPpT4_HTX1-PARS1-HsCas9-Gen

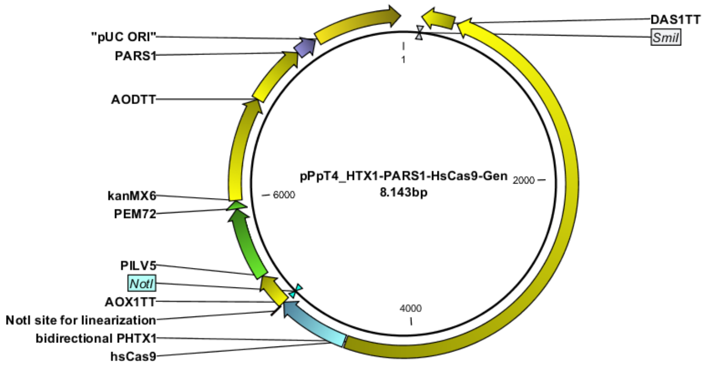

pJet-GUT1-PARS1

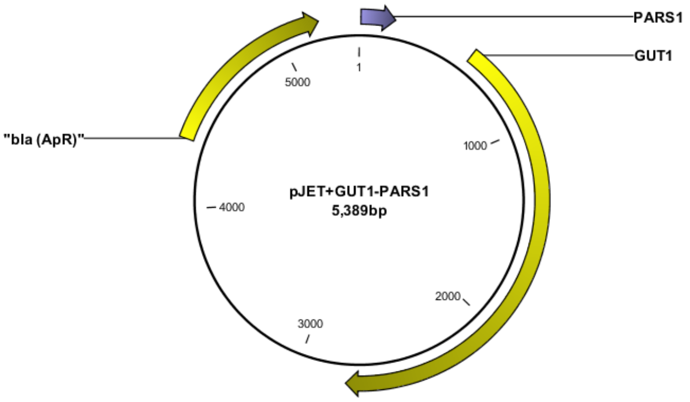

S3: Sequencing of the *GUT1* locus upon CRISPR/Cas9 cleavage in *P. pastoris Δku70*

When solely a CRISPR/Cas9 plasmid was transformed to target the *GUT1* gene (no donor), sequencing of *P. pastoris Δku70, Δgut1* CRISPR/Cas9 transformants resulted in ~600 bp deletions upstream of the Cas9 cleavage site (Figure S3B,C). Compared to the *P. pastoris Δku70* transformants, *P. pastoris* wildtype *Δgut1* transformants bore short insertions or deletion mutations (indels, Weninger et al., (2016)) (Figure S3B).

The co-transformation of CRISPR/Cas9 plasmids and donor DNA, and sequencing of *P. pastoris Δku70 Δgut1* transformants resulted either in the complete removal of the *GUT1* gene or sequencing reads, which could only be aligned partially, containing mixed sequences. These mixed reads may indicate chromosomal rearrangements or translocations. An example is shown in Figure S3D.

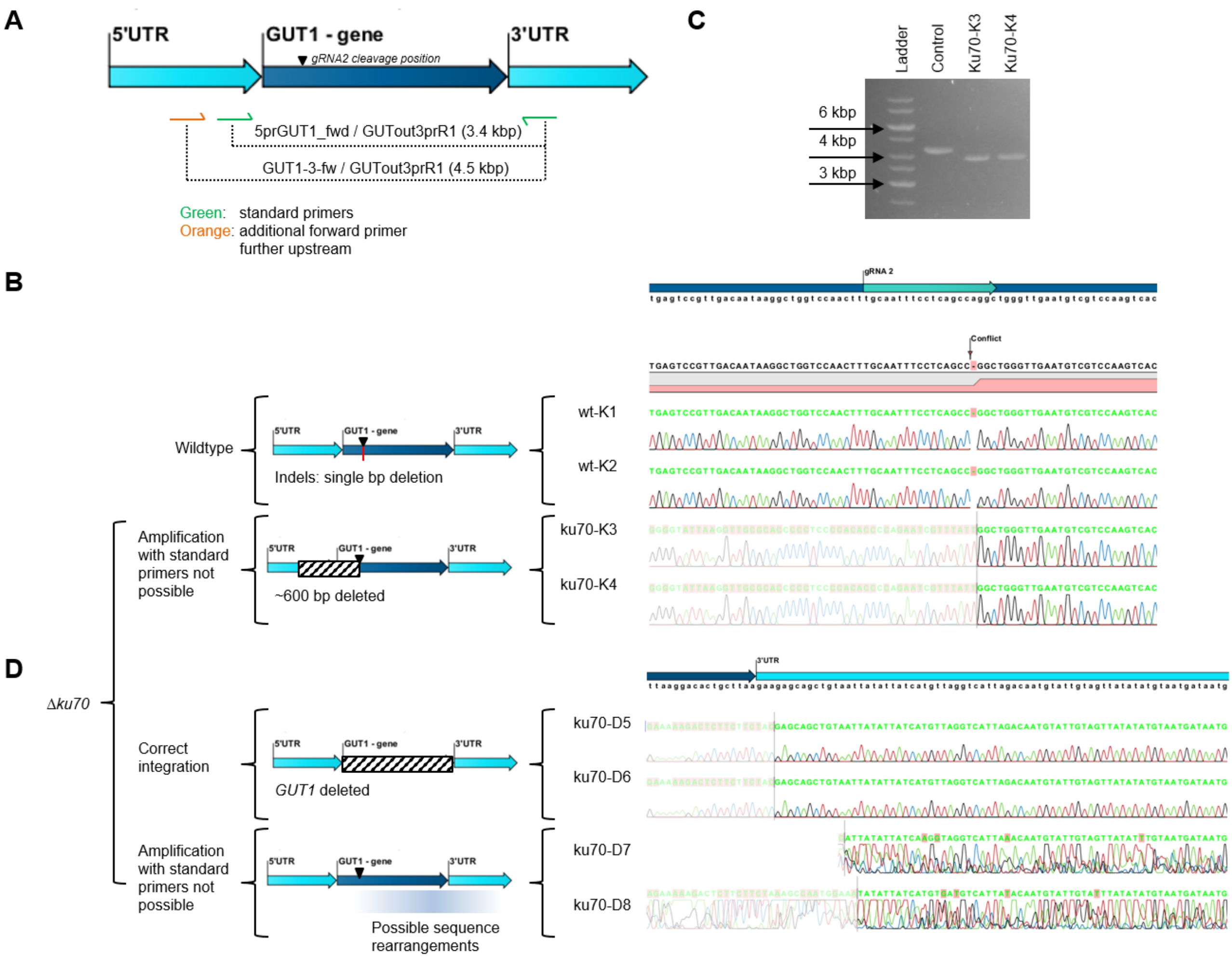

S 3: Sequencing of the *GUT1* locus of *P. pastoris Δku70* transformants. A. Scheme of the *GUT1* locus and primer pairs used for PCR amplification/sequencing. **B.** Sequencing of the *GUT1* locus of *P. pastoris* wildtype and *Δku70* transformants (transformed with CRISPR/Cas9 plasmids to target *GUT1* [without donor DNA]). NHEJ-mediated repair in *P. pastoris* wildtype resulted in short indel mutations (wt-K1 and wt-K2). *P. pastoris Δku70 Δgut1* transformants ku70-K3 and ku70-K4 bore large deletion mutation (approximately 600 bp). The region upstream of the Cas9 cleavage site was deleted. **C.** Also the PCR products (primers GUT1-3-fw and GUTout3prR1) of ku70-K3 and ku70-K4 were ca. 0.6 kbp reduced in size compared to the wildtype control, in line with the deletions observed in the sequencing. **D.** Sequencing of the *GUT1* locus of *P. pastoris Δku70* transformants, which were co-transformed with CRISPR/Cas9 plasmids to target *GUT1* and donor DNA. The sequence of the 3' end of *GUT1* and 3'UTR aligned to various sequencing reads is shown. ku70-D5 and ku70-D6: the integration of the donor fragment for the removal for the *GUT1* CDS was obtained; the shaded part of the sequence corresponds to the 5'UTR of *GUT1*. Ku70-D7 and ku70-D8: If no donor was integrated mixed reads were obtained, which may indicate sequential rearrangements.
